# Importance of Structural Relaxation on the Electrical Characteristics and Bias Stability of Solution-Processed ZnSnO Thin-Film Transistors

**DOI:** 10.3390/nano12183097

**Published:** 2022-09-07

**Authors:** Yu-Jin Hwang, Do-Kyung Kim, Sang-Hwa Jeon, Ziyuan Wang, Jaehoon Park, Sin-Hyung Lee, Jaewon Jang, In Man Kang, Jin-Hyuk Bae

**Affiliations:** 1 School of Electronic and Electrical Engineering, Kyungpook National University, 80 Daehakro, Bukgu, Daegu 41566, Korea; 2 Department of Electronic Engineering, Hallym University, Chuncheon 24252, Korea; 3 School of Electronics Engineering, Kyungpook National University, 80 Daehakro, Bukgu, Daegu 41566, Korea

**Keywords:** structural relaxation, electrical characteristics, bias stability, zinc–tin oxide, thin-film transistor, solution process

## Abstract

Effect of structural relaxation (SR) on the electrical characteristics and bias stability of solution-processed zinc–tin oxide (ZTO) thin-film transistors (TFTs) were systematically investigated by controlling the annealing time of the ZTO semiconductor films. Note that SR was found to increase with increased annealing time. Due to the increased SR, the ratio of oxygen vacancies (V_O_) increased from 21.5% to 38.2%. According to increased V_O_, the mobility in the saturation region was exhibited by a sixfold increase from 0.38 to 2.41 cm^2^ V^−1^ s^−1^. In addition, we found that the threshold voltage negatively shifted from 3.08 to −0.95 V. Regarding the issue of bias stability, according to increased SR, positive-bias stress of the ZTO TFTs was enhanced, compared with reverse features of negative-bias stress. Our understanding is expected to provide a basic way to improve the electrical characteristics and bias stability of rare-metal-free oxide semiconductor TFTs, which have not been sufficiently studied.

## 1. Introduction

Oxide thin-film transistors (TFTs) are widely utilized as key unit devices in displays and sensors due to their unique advantages such as optical transparency, outstanding electrical characteristics, and mechanical flexibility [1,2,3,4,5]. Recently, the solution process technique has received considerable attention because it allows for the processing of a large area at a low cost compared with conventional vacuum processes such as sputtering [6,7,8]. However, the electrical characteristics and stability of solution-processed oxide TFTs are poor compared with those of vacuum-process-based thin films [9,10,11]. This is because the thin films produced by the solution process exhibit physical defects, such as pinholes and pore sites, as well as other chemical defects, and low film uniformity [12,13]. For example, in solution-processed organic or oxide semiconductor devices, the grain size, grain boundary, structural disorder, and molecular alignment characteristics of semiconductors could yield the physical or chemical defects and limit the charge transport [14,15,16]. These undesirable results cause the degradation of electrical characteristics and device-to-device uniformity. Therefore, it is necessary to improve the electrical characteristics and stability of solution-processed oxide TFTs. Unfortunately, a trade-off relationship may exist between the high mobility and stability of oxide TFTs [17,18,19]. For example, high-mobility oxide TFTs with high hydrogen contents exhibit relatively inferior negative-bias temperature-stress-induced stability compared with low-mobility oxide TFTs [19]. This is because mobility improvement originates from the increase in the concentration of oxygen vacancies (V_O_) or hydrogen, which can act as defects under specific stress conditions [20,21]. Therefore, simultaneous considerations of the electrical characteristics and stability are required to optimize and utilize oxide TFTs as unit devices in electronic applications. From this perspective, the structural relaxation (SR) of oxide semiconductors is a significant factor in improving and optimizing the characteristics of oxide-semiconductor-based TFTs. SR is a process that involves the formation of a more stable structure by reducing the degree of structural disorder by reorganizing atomic components, such as oxygen anions, metal cations, V_O_, and physical or chemical defects in metal oxide films [22,23]. Understanding and controlling this phenomenon is essential because it directly affects the electrical characteristics and stability of devices.

Indium–gallium–zinc oxide (IGZO) has been widely employed as an oxide semiconductor material for TFTs in the display industry [24,25,26]. IGZO is a desirable semiconductor material for the mass production of display panels as it exhibits high device-to-device uniformity due to its amorphous phase and enables elaborate control of the electrical characteristics and reliability of TFTs through the adjustment of indium and gallium concentrations. As a large high-resolution area is required, it is crucial to enhance the mobility while maintaining a low cost. However, indium and gallium are rare metals, and mobility improvement is limited, due to the inclusion of gallium. Consequently, rare-metal-free oxide semiconductors have recently received considerable attention for low-cost, high-mobility oxide TFTs [27,28]. Zinc–tin oxide (ZTO) is a representative rare-metal-free oxide semiconductor due to its amorphous phase and the incorporation of tin, which is advantageous in terms of cost reduction and which results in an increase in the carrier concentration of semiconductors. However, solution-processed ZTO is known to exhibit poor electrical characteristics and bias stability compared with sputtered oxide semiconductors. Thus, an in-depth understanding of the SR phenomenon is required. To that end, research on the effects of SR on solution-processed ZTO semiconductors should be conducted to obtain high-performance and stable oxide TFTs.

In this study, we investigated the effects of SR on the electrical characteristics and stability of solution-processed ZTO TFTs. The phenomenon of SR was found to accelerate with increased annealing time of the ZTO semiconductor. By conducting thin-film analysis, the physical and chemical changes in ZTO TFTs resulting from SR were demonstrated. As the SR was improved via the oxygen vacancy generation mechanism in the ZTO semiconductor, the saturation mobility (μ_sat_) was enhanced, and V_T_ shifted negatively. Furthermore, the negative-bias stress stability related to the oxygen vacancies deteriorated. In contrast, as the SR was improved, the number of localized states near the conduction band of ZTO decreased due to the reduction in free volume and stabilization of the local atomic condition; thus, the positive-bias stress stability was improved.

## 2. Materials and Methods

A ZTO precursor solution was prepared for the fabrication of the semiconductor thin films. A 0.17 M ZTO solution was synthesized by dissolving zinc chloride and tin (II) chloride dihydrate (Sigma-Aldrich, Saint Louis, MO, USA) in 2-methoxyethanol. The zinc-to-tin molar ratio was 6:4. The solution was stirred at room temperature for 30 min to obtain a homogeneous solution. To fabricate the ZTO TFTs, 100 nm-thick SiO_2_ was grown via a thermal oxidation process on a Si wafer for the gate dielectric. The SiO_2_/p^++^ Si wafer substrates were cleaned via sonication for 10 min each in acetone, isopropyl alcohol, and deionized water. The Si/SiO_2_ wafer substrates were also cleaned by sonication for 10 min each in acetone, isopropanol, and deionized water and then dried on a hot plate in air to remove residual moisture. The clean substrates were exposed to ultraviolet (UV) light for 20 min in ambient air to improve their surface wettability. The prepared ZTO precursor solution was spin-coated at 4000 rpm for 12 s onto a UV-exposed Si/SiO_2_ wafer. For solidification of the ZTO semiconductors, ZTO-coated wafers were pre-annealed at 110 °C for 30 min. Subsequently, they were post-annealed at 500 °C for 10–40 min to investigate the SR effects. The ZTO thin films annealed for 10, 20, 30, and 40 min were labelled as SR-10, -20, -30, and -40, respectively. Subsequently, 50 nm-thick Al source and drain electrodes were deposited using thermal evaporation. The channel length (L) and width (W) were 100 μm and 1000 μm, respectively. Finally, the ZTO TFTs were vacuum treated in a vacuum oven at 140 °C for 50 min to improve their switching characteristics.

Transmission electron microscopy (TEM) Titan G2 ChemiSTEM Cs Probe (FEI Company, Hillsboro, OR, USA) was used to measure the film thickness. The chemical compositions of the ZTO thin films were investigated using X-ray photoelectron spectroscopy (XPS) NEXSA (Thermo Fisher Scientific, Waltham, MA, USA) with an Al Kα (1486.6 eV) light source. The electrical characteristics of the ZTO TFTs were analyzed using a probe station Model 4000 (MS Tech, Hwaseong, Korea) equipped with a precision LCR meter (Keysight, Santa Rosa, CA, USA) and semiconductor parameter analyzer Keithley 2636 B (Tektronix, Inc., Beaverton, OR, USA). All the specimens were analyzed immediately after fabrication at room temperature in a dark environment.

## 3. Results and Discussion

### 3.1. Physical and Chemical Properties of Structurally Relaxed ZTO Thin Films

**Figure 1:**
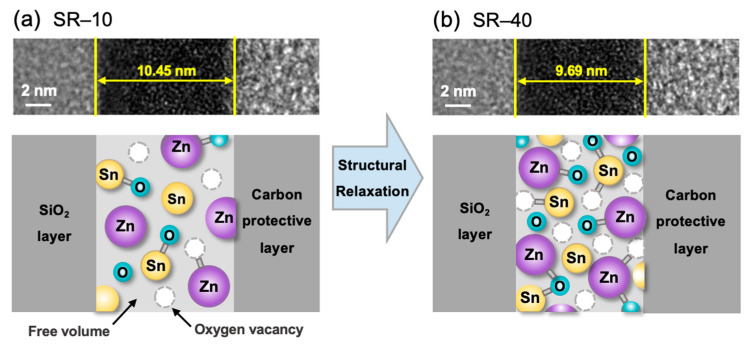
TEM images (**top**) and corresponding schematics (**bottom**) for (**a**) SR-10 and (**b**) SR-40. The schematics illustrate the change produced in the atomic structure by structural relaxation.

Figure 1a,b illustrates the TEM images (top) and corresponding schematic illustrations (bottom) of SR-10 and SR-40 to discuss the effects of SR on the ZTO thin films, respectively. The ZTO film thicknesses of SR-10 and SR-40 were 10.45 nm and 9.69 nm, respectively. This is because the SR effect was enhanced during additional thermal annealing of the ZTO thin film. Generally, during the thermal annealing process, the atoms within a semiconductor film are reorganized to minimize the degree of structural disorder and free volume in the oxide semiconductors. Furthermore, by annealing at temperatures lower than 600 °C, ZTO can reduce the density of the film via densification while maintaining the amorphous structure of the film [23]. Particularly, SR can contribute to a decrease in the free volume and an increase in the film density and carrier concentration. It is known that the densification by thermal annealing is significantly affected by annealing temperature rather than annealing time [22]. However, in this study, as the annealing time increased by 30 min, the thickness of the ZTO film decreased by about 7%, and the results suggest that the annealing time also affects the densification of the ZTO film. The detailed effects of SR on the film properties and electrical characteristics of TFTs will be discussed later.

**Figure 2:**
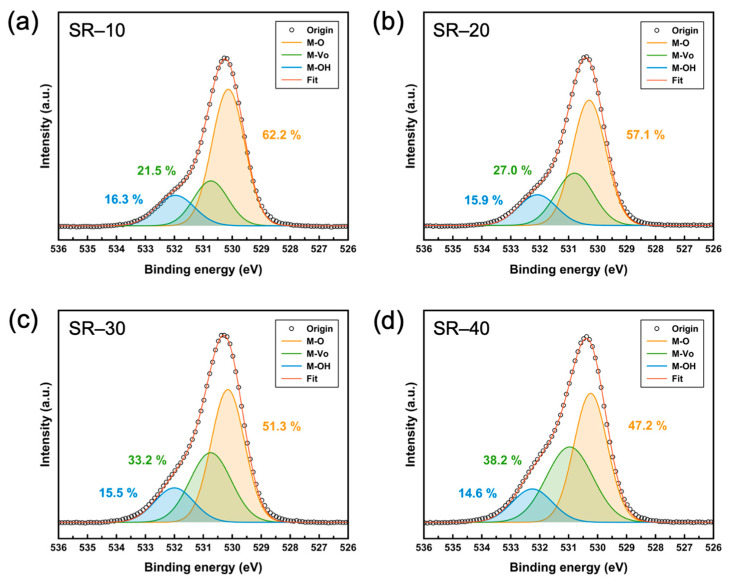
XPS O 1s spectra for (**a**) SR-10, (**b**) SR-20, (**c**) SR-30, and (**d**) SR-40.

XPS analysis was performed to demonstrate the effect of SR on the atomic composition of ZTO semiconductors. Figure 2a–d illustrate deconvoluted XPS O 1s spectra of specimens from SR-10 to -40, respectively. The three primary peaks, positioned at ~530.2, ~530.9, and ~532.2 eV, correspond to metal–oxygen (M–O), V_O_, and metal hydroxide (M–OH), respectively. The M–O ratio of the ZTO semiconductor decreased from 62.2% to 47.2%, whereas the V_O_ ratio increased from 21.5% to 38.2% as the annealing time of ZTO increased. This result indicates that the ratio of V_O_ in the oxide semiconductor increases due to the increased SR, as depicted in Figure 1. In particular, the M–OH ratio decreased from 16.3% to 14.6%. It should be noted that M–OH groups in oxide semiconductors can act as trap sites and limit the charge transport of carriers [29]. Consequently, increasing the annealing time of the solution-processed ZTO semiconductor can enhance the film density and contribute to an increase in the carrier concentration of semiconductors. These physical and chemical changes in semiconductor thin films resulting from SR can induce variations in the electrical characteristics and stability of ZTO TFTs.

### 3.2. Effects of Structural Relaxation on Electrical Characteristics in ZTO TFTs

**Figure 3:**
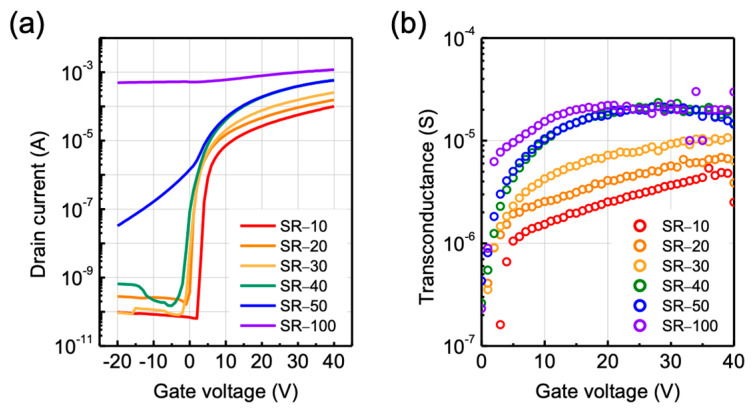
(**a**) Transfer characteristics and (**b**) transconductance of ZTO TFTs with various annealing times.

ZTO TFTs with different annealing times were fabricated to demonstrate the effect of SR on their electrical characteristics and bias stability. Figure 3a,b depict the transfer characteristics and transconductance (g_m_) of ZTO TFTs with different annealing time. In Figure 3a, it can be seen that the negative shift of the threshold voltage (V_T_) and the increase in the on current are increased as the annealing time is increased. As the annealing time increases, the carrier concentration also increases due to the additional formation of oxygen vacancy which play as a donor. Thus, electrons are easily accumulated by positive gate voltage at the dielectric–semiconductor interface. However, a long annealing time of 50 min or more increases the off current and subthreshold swing, leading to deterioration of the switching characteristics. This is because the formation of excessive oxygen vacancies, due to a long annealing time, sharply increases the carrier concentration and induces a decrease in metal–oxygen bonding, thereby degrading the switching and charge transport properties. Detail electrical characteristics of SR-10–SR-40 such as saturation mobility, threshold voltage, subthreshold swing, and hysteresis will be discussed in the next paragraph. The g_m_ was extracted to estimate the charge transport mechanism of ZTO TFTs with various annealing time. Unlike single-crystal Si-based MOSFETs, the charge transport mechanism in oxide semiconductor systems is generally based on trap-limited and percolation conduction [30,31]. This unique charge transport mechanism is due to the structural disorder and combination of the metal cations in the oxide semiconductor [30,31]. Therefore, as the Fermi level located above the conduction band minimum increases with an increase in the gate voltage in the oxide TFT, the potential barrier above the conduction band minimum appears to decrease [16]. These changes lead to a decrease in the percolation effect in the conduction of the electrons. Accordingly, in the oxide semiconductor system, a phenomenon in which the transconductance gradually increases as the gate voltage increases can be observed because of the percolation effect. Due to the origin of the conduction mechanism, oxide semiconductor TFTs with high carrier concentration have a relatively high Fermi level, such that as the gate voltage increases, the scattering effect more dominantly affects the charge transport than the percolation effect. From SR-10 to SR-30, transconductance increases as the gate voltage increases, thereby implying that the percolation conduction is mainly achieved in ZTO TFTs with a relatively short annealing time. However, from SR-40, transconductance becomes saturated at high gate voltage. It indicates that interface scattering became dominant in the devices with a long annealing time which have a high carrier concentration.

**Figure 4:**
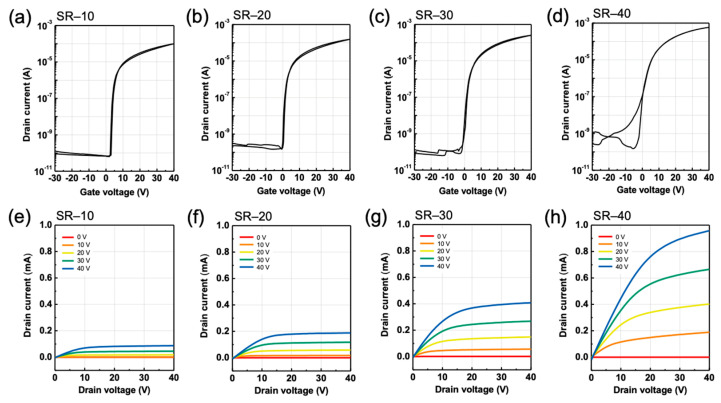
Transfer characteristics of ZTO TFT specimens: (**a**) SR-10, (**b**) SR-20, (**c**) SR-30, and (**d**) SR-40. Output characteristics of ZTO TFT specimens: (**e**) SR-10, (**f**) SR-20, (**g**) SR-30, and (**h**) SR-40.

Figure 4 depict the transfer (a–d) and output characteristics (e–h) of the solution-processed ZTO TFT specimens SR-10–SR-40. A drain voltage (V_D_) of 40 V was used to record the transfer characteristics. In addition, a gate voltage (V_G_) in the range of 0–40 V was applied to record the output characteristics. As the SR increased from 10 to 40 min, V_T_ negatively shifted from 3.08 V to −0.95 V, and μsat increased from 0.38 cm^2^ V^−1^ s^−1^ to 2.41 cm^2^ V^−1^ s^−1^. μ_sat_ values for the ZTO TFTs were calculated using the following equation:

(1) $$\mu_{\mathrm{sat}}=\frac{2L}{{\mathrm{WC}}_{i}}\cdot {\left(\frac{\partial \sqrt{I_{D,\mathrm{sat}}}}{\partial V_{G}}\right)}^{2}$$

where C_i_ and I_D,sat_ denote the capacitance per unit area and the drain current in the saturation regime, respectively. In addition, the subthreshold swing (SS) and on/off ratio (Ion/off) were increased from 0.36 V dec^−1^ to 1.25 V dec^−1^ and from 1.58 × 10^6^ to 3.76 × 10^6^, respectively. This significant change in the electrical characteristics is related to the carrier concentration. As SR increases, the Vo ratio of ZTO increases, as illustrated in Figure 2.

Therefore, electron concentration in the semiconductor is increased due to the free carriers generated by Vo with the increase in SR. As the number of free electrons increases, they can easily be collected under the effect of V_G_, thereby inducing a negative V_T_ and high I_on_ and μ_sat_. The higher SS of the ZTO TFT specimen SR-40 compared with that for SR-10 is attributed to the diffusion of oxygen during the additional annealing process [32]. The electrical parameters of SR-10–SR-40 are summarized in Table 1.

### 3.3. Effects of Structural Relaxation on Bias-Induced Instabilities in ZTO TFTs

**Figure 5:**
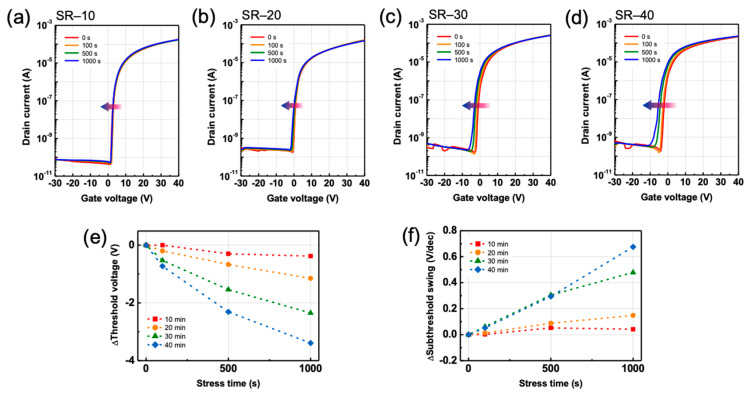
Transfer characteristics as a function of a negative-bias stress time for ZTO TFT specimens: (**a**) SR-10, (**b**) SR-20, (**c**) SR-30, and (**d**) SR-40. (**e**) Threshold voltage and (**f**) subthreshold swing shift of ZTO TFTs for a negative-bias stress.

It is important to understand the electrical stability of oxide TFTs, because the variations in electrical characteristics, such as VT and SS, by the bias stress limit the utilization of oxide TFTs in display applications. The bias-stress-induced instability in ZTO TFTs was investigated by applying both positive and negative biases for time durations of 0, 100, 500, and 1000 s at room temperature in a dark environment. Negative-bias stress (NBS) and positive-bias stress (PBS) tests were conducted at V_G_ = −30 V and V_G_ = +30 V, respectively. A V_D_ of +40 V was applied for both the NBS and PBS tests. Figure 5a–d illustrate the transfer characteristics of ZTO TFTs with SR enhancement as a function of the NBS duration. Figure 5e presents ∆V_T_ values ranging from 0 to 1000 s in the NBS test. The negative-bias-induced instability gradually increased as the SR effect improved. The ∆V_T_ values for SR-10–SR-40 were −0.38, −1.15, −2.35, and −3.39 V, respectively. This is attributed to the increase in V_O_ of ZTO due to SR. Under a strong negative gate bias condition, band bending becomes extremely severe. Consequently, the Fermi energy level approaches the valence band at the gate dielectric–semiconductor interface, and the formation energy of V_O_^2+^ decreases [33]. V_O_ is transformed to V_O_^2+^ by the release of two free electrons, which contribute to the carrier concentration. Thus, V_T_ shifts in the negative direction as the stress duration increases. Here, hole trapping at the semiconductor–dielectric interface and hole injection at the gate dielectric could also be considered as the origins of the negative shift in V_T_ for NBS [34]. However, the SS increased as the stress duration increased for all ZTO TFTs, as illustrated in Figure 5f. In addition, ∆SS increased from 0.042 V dec^−1^ to 0.676 V dec^−1^ as SR increased. An increase in SS can be interpreted as an increase in the number of trap sites (N_T_), according to the following equation:

(2) $$N_{T}=\left[\frac{\mathrm{SS}\log\left(e\right)}{\frac{\mathrm{kT}}{q}}-1\right]\frac{C_{i}}{q}$$

where q, k, and T denote the electron charge, Boltzmann constant, and absolute temperature, respectively. Therefore, the increase in SS indicates that the NBS-induced instability is attributed not to the charge trapping or injection mechanism, but to the defect creation mechanism [34,35]. Here, V_O_^2+^, which are generated under the effect of a high negative gate bias, migrate to the dielectric–semiconductor interface under the effect of the negative gate bias and act as acceptor-like traps at the interface [34,35]. V_O_^2+^ accept free electrons when the devices are turned on, thereby yielding a high SS in the ZTO TFTs. The defect-creation mechanism is also consistent with the increase in V_O_, resulting from the improvement in SR, which is demonstrated through the XPS analysis, as illustrated in Figure 2.

**Figure 6:**
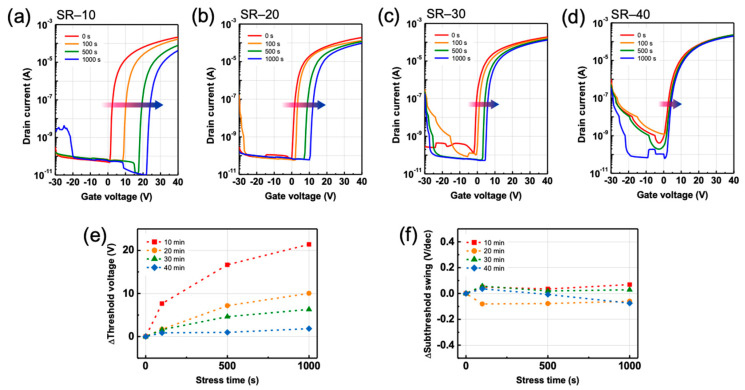
Transfer characteristics as a function of a positive-bias stress time for ZTO TFT specimens: (**a**) SR-10, (**b**) SR-20, (**c**) SR-30, and (**d**) SR-40. (**e**) Threshold voltage and (**f**) subthreshold swing shift of ZTO TFTs for a positive-bias stress.

Figure 6a–d depict the transfer characteristics of ZTO TFTs with SR enhancement as a function of the PBS duration. In the PBS test, ∆VT was gradually decreased from 21.4 to 1.84 V as SR was enhanced, as illustrated in Figure 6e. The SS of the ZTO TFTs was extracted to reveal the mechanism responsible for the shift in VT. For all ZTO TFTs, regardless of the ZTO condition, ∆SS appears negligible as the stress duration increases, as confirmed in Figure 6f. This contrasts with the results obtained from the NBS test. The fact that SS does not significantly change with an increase in the stress duration suggests that additional defects are not created in the semiconductor or at the semiconductor–dielectric interface. Furthermore, considering that oxide semiconductors are n-type semiconductors wherein electrons are the majority carriers, electrons can easily be trapped at the semiconductor–dielectric interface or easily injected into the SiO_2_ dielectric under positive gate bias conditions. Therefore, the negligible change in SS with increasing stress duration indicates that the positive-bias-induced instability in ZTO TFTs is caused not by defect creation mechanisms, such as the double ionization of oxygen vacancies, but by electron trapping at the semiconductor–dielectric interface or electron injection at the gate dielectric. In addition, the densification of ZTO by SR over a long period may reduce the localized tail states, which act as acceptor-like traps, under the conduction band minimum [22,31].

## 4. Conclusions

The effects of SR on the electrical characteristics and bias instability of solution-processed ZTO TFTs were investigated by controlling the annealing time. The ratio of V_O_ for SR-10, -20, -30, and -40 increased to 21.5, 27.0, 33.2, and 38.2%, respectively, under the SR effect. Due to the increased carrier concentration, μ_sat_ increased from 0.38 cm^2^ V^−1^ s^−1^ to 2.41 cm^2^ V^−1^ s^−1^, and V_T_ negatively shifted from 3.08 V to −0.95 V. In addition, as SR was improved, SS increased from 0.36 V dec^−1^ to 1.25 V dec^−1^. In particular, the NBS stability associated with V_O_ deteriorated from −0.38 V (SR-10) to 3.39 V (SR-40) via the generation and migration of V_O_^2+^. Meanwhile, as the SR effect improved, the number of localized states near the conduction band of ZTO decreased due to a reduction in the free volume and stabilization of the local atomic condition; thus, the PBS stability of ZTO TFTs improved from 21.4 V (SR-10) to 1.84 V (SR-40). These results are expected to contribute to the optimization of electrical characteristics and stability of rare-metal-free oxide TFTs.

## Figures and Tables

**Table 1:** Summary of electrical parameters of TFT specimens: SR-10–SR-40.

SR	μ_sat_ (cm^2^ V^−1^ s^−1^)	V_T_ (V)	SS (V dec^−1^)	Ion/off Ratio
10	0.38	3.08	0.36	1.58 × 10^6^
20	0.50	0.45	0.45	1.06 × 10^6^
30	1.10	0.15	0.83	4.07 × 10^6^
40	2.41	−0.95	1.25	3.9 × 10^6^

## Data Availability

Not applicable.
